# Supplementary material for: Prostatic urinary tract visualization with super-resolution deep learning models
Source: PLoS One. 2023 Jan 6;18(1):e0280076. doi: 10.1371/journal.pone.0280076 (PMC9821403; doi:10.1371/journal.pone.0280076)
Supplement: S1 File — (DOCX) [file pone.0280076.s001.docx]

Supporting information with our minimal data set of urethra’s visibility score

| Model title | | PU-MRI [8] | | EDSR [21] | | WDSR [23] | | SRGAN [25] | | RDN [26] | | CT  without catheter [8] | | CT  with catheter [8] | |
| --- | --- | --- | --- | --- | --- | --- | --- | --- | --- | --- | --- | --- | --- | --- | --- |
| Operator | | 1 | 2 | 1 | 2 | 1 | 2 | 1 | 2 | 1 | 2 | 1 | 2 | 1 | 2 |
| Case No. | 1 | 4 | 5 | 2 | 4 | 2 | 5 | 2 | 5 | 4 | 5 | 1 | 1 | 5 | 5 |
|  | 2 | 5 | 5 | 4 | 5 | 3 | 4 | 3 | 4 | 5 | 5 | 1 | 1 | 5 | 5 |
|  | 3 | 2 | 3 | 2 | 3 | 2 | 3 | 2 | 4 | 3 | 4 | 1 | 1 | 5 | 5 |
|  | 4 | 2 | 4 | 2 | 4 | 2 | 4 | 2 | 4 | 3 | 4 | 1 | 1 | N.A. | N.A. |
|  | 5 | 3 | 3 | 2 | 4 | 3 | 4 | 3 | 4 | 3 | 4 | 1 | 1 | 5 | 5 |
|  | 6 | 4 | 4 | 3 | 4 | 3 | 4 | 3 | 4 | 4 | 4 | 1 | 1 | 5 | 5 |
|  | 7 | 4 | 4 | 3 | 4 | 3 | 4 | 3 | 4 | 4 | 5 | 1 | 1 | 5 | 5 |
|  | 8 | 5 | 3 | 3 | 3 | 4 | 3 | 4 | 3 | 5 | 3 | 1 | 1 | 5 | 5 |
|  | 9 | 4 | 4 | 3 | 4 | 3 | 4 | 3 | 4 | 5 | 5 | 1 | 1 | 5 | 5 |
|  | 10 | 3 | 3 | 2 | 3 | 2 | 3 | 2 | 4 | 3 | 4 | 1 | 1 | 5 | 5 |
|  | 11 | 3 | 4 | 3 | 4 | 3 | 4 | 3 | 4 | 4 | 4 | 1 | 1 | 5 | 5 |
|  | 12 | 2 | 3 | 2 | 3 | 2 | 3 | 2 | 3 | 2 | 3 | 1 | 1 | 5 | 5 |
|  | 13 | 4 | 4 | 2 | 4 | 3 | 4 | 3 | 4 | 4 | 5 | 1 | 1 | N.A. | N.A. |
|  | 14 | 3 | 4 | 4 | 4 | 4 | 5 | 4 | 4 | 4 | 5 | 1 | 1 | 5 | 5 |
|  | 15 | 5 | 5 | 5 | 4 | 5 | 4 | 5 | 4 | 5 | 5 | 1 | 1 | N.A. | N.A. |
|  | 16 | 1 | 4 | 1 | 4 | 1 | 4 | 1 | 3 | 2 | 4 | 1 | 1 | N.A. | N.A. |
|  | 17 | 3 | 3 | 2 | 3 | 2 | 4 | 2 | 4 | 2 | 4 | 1 | 1 | 5 | 5 |
|  | 18 | 5 | 3 | 3 | 4 | 3 | 4 | 3 | 4 | 5 | 5 | 1 | 1 | N.A. | N.A. |
|  |  |  |  |  |  |  |  |  |  |  |  |  |  |  |  |
|  | 19 | 4 | 3 | 3 | 2 | 3 | 2 | 3 | 3 | 3 | 4 | 1 | 1 | 5 | 5 |
|  | 20 | 4 | 3 | 3 | 3 | 3 | 3 | 3 | 3 | 4 | 4 | 1 | 1 | 5 | 5 |
|  | 21 | 4 | 4 | 3 | 4 | 3 | 3 | 3 | 3 | 4 | 4 | 1 | 1 | N.A. | N.A. |
|  | 22 | 3 | 4 | 2 | 4 | 2 | 4 | 2 | 4 | 3 | 5 | 1 | 1 | 5 | 5 |
|  | 23 | 3 | 3 | 2 | 4 | 2 | 4 | 2 | 4 | 3 | 5 | 1 | 1 | 5 | 5 |
|  | 24 | 3 | 4 | 3 | 4 | 3 | 4 | 3 | 4 | 4 | 5 | 1 | 1 | 5 | 5 |
|  | 25 | 3 | 3 | 2 | 3 | 2 | 3 | 2 | 3 | 3 | 4 | 1 | 1 | 5 | 5 |
|  | 26 | 5 | 4 | 3 | 4 | 2 | 4 | 2 | 4 | 5 | 5 | 1 | 1 | 5 | 5 |
|  | 27 | 3 | 4 | 2 | 3 | 2 | 3 | 2 | 3 | 3 | 4 | 1 | 1 | 5 | 5 |
|  | 28 | 4 | 3 | 3 | 3 | 3 | 3 | 3 | 3 | 4 | 4 | 1 | 1 | 5 | 5 |
|  | 29 | 4 | 4 | 3 | 4 | 3 | 4 | 3 | 4 | 4 | 5 | 1 | 1 | 5 | 5 |
|  | 30 | 4 | 4 | 4 | 4 | 4 | 3 | 4 | 3 | 5 | 4 | 1 | 1 | 5 | 5 |

PU-MRI, post urination magnetic resonance imaging; EDSR, enhanced deep super resolution network; WDSR, wide activation for efficient and accurate image super resolution network; SRGAN, photo-realistic single image super resolution using a generative adversarial network; RDN, residual dense network, CT: computed tomography, and N.A: not applicable (The target patients were unable to insert a urethral catheter for some reason, such as pain during insertion).
