# Supplementary material for: Prostatic urinary tract visualization with super-resolution deep learning models
Source: PLoS One. 2023 Jan 6;18(1):e0280076. doi: 10.1371/journal.pone.0280076 (PMC9821403; doi:10.1371/journal.pone.0280076)
Supplement: Attachment — Submitted filename: For_reply_20221121.docx [file pone.0280076.s003.docx]

We would like to thank all of the editors and reviewers for their valuable comments, which have helped us to substantially improve our manuscript. Our point-by-point responses to the comments are presented below.

***Reviewer #1 comments***

*I would like to thank the authors for thoroughly addressing my comments and providing further insights. I have a few minor comments remaining:*

1. *Please clarify in the manuscript that this is a pure retrospective study and that therefore for inference HR images were downsampled in the image domain.*

**Response**: We thank the reviewer for the comment. We have revised the text as follows.

**Patients** (Pages 8, Lines 115–118)

This retrospective study included a total of 900 images of 30 patients with prostate cancer who had previously undergone real-time-image gated spot-scanning proton therapy (RGPT) using three inserted fiducial markers at our institution from October 2019 to October 2020.

**Super-resolution deep learning model** (Pages 12, Lines 163–164)

Each LR image was obtained from the HR DIV2K image by bicubic downscaling in the image domain.

2. *Please report the amount of trainable parameters for each network.*

**Response**: We thank the reviewer for the comment. We have revised the text as follows.

**Image acquisition** (Page 12–13, Lines 170–178)

The DIV2K dataset comprises 800 training images, 100 validation images, 100 test images, and bicubic downsampled images. In all SR deep learning models, we train with Adam optimizer [27] by setting $\beta_{1}=0.9$, $\beta_{2}=0.999$, and $\epsilon={10}^{-8}$. The number of epochs was 50; batch size, 16; and learning rate$, {10}^{-4}$. The loss functions for the SR deep learning models were L1 loss for EDSR, WDSR, and RDN; and VGG54 content loss for SRGAN. The trainable parameters for each network were 43 M, 0.62 M, 1.55 M, and 22 M for EDSR, WDSR, SRGAN, and RDN, respectively. In this study, these architectures were not modified further and were used as proposed in the respective references [21, 23, 25, 26]. For testing, we use 900 PU-MRI images of 30 patients.

3. *Please report the amount of test images used for further analysis.*

**Response**: We thank the reviewer for the comment. We have included the information per your comment as follows.

**Image acquisition** (Page 12–13, Lines 170–178)

The DIV2K dataset comprises 800 training images, 100 validation images, 100 test images, and bicubic downsampled images. In all SR deep learning models, we train with Adam optimizer [27] by setting $\beta_{1}=0.9$, $\beta_{2}=0.999$, and $\epsilon={10}^{-8}$. The number of epochs was 50; batch size, 16; and learning rate$, {10}^{-4}$. The loss functions for the SR deep learning models were L1 loss for EDSR, WDSR, and RDN; and VGG54 content loss for SRGAN. The trainable parameters for each network were 43 M, 0.62 M, 1.55 M, and 22 M for EDSR, WDSR, SRGAN, and RDN, respectively. In this study, these architectures were not modified further and were used as proposed in the respective references [21, 23, 25, 26]. For testing, we use 900 PU-MRI images of 30 patients.

.

4. *L314: Please correct typo “described”*

**Response**: We thank the reviewer for the comment. We have revised the sentence as follows.

**Image acquisition** (Page 22, Line 317)

Knoll et al. described the risk of using a database of natural scene images for transfer learning in medical images [34].
